# Expression Silencing of Mitogen-Activated Protein Kinase 8 Interacting Protein-1 Conferred Its Role in Pancreatic β-Cell Physiology and Insulin Secretion

**DOI:** 10.3390/metabo13020307

**Published:** 2023-02-20

**Authors:** Rania Saeed, Abdul Khader Mohammed, Sarra E. Saleh, Khaled M. Aboshanab, Mohammad M. Aboulwafa, Jalal Taneera

**Affiliations:** 1 Department of Microbiology and Immunology, Faculty of Pharmacy, Ain Shams University, Cairo 11566, Egypt; 2 Sharjah Institute for Medical Research, University of Sharjah, Sharjah 27272, United Arab Emirates; 3 Faculty of Pharmacy, King Salman International University, Ras-Sudr 46612, Egypt; 4 Department of Basic Sciences, College of Medicine, University of Sharjah, Sharjah 27272, United Arab Emirates

**Keywords:** MAPK8IP1, insulin secretion, INS-1 cells, human islets, type 2 diabetes

## Abstract

Mitogen-activated protein kinase 8 interacting protein-1 (MAPK8IP1) gene has been recognized as a susceptibility gene for diabetes. However, its action in the physiology of pancreatic β-cells is not fully understood. Herein, bioinformatics and genetic analyses on the publicly available database were performed to map the expression of the *MAPK8IP1* gene in human pancreatic islets and to explore whether this gene contains any genetic variants associated with type 2 diabetes (T2D). Moreover, a series of functional experiments were executed in a rat insulinoma cell line (INS-1 832/13) to investigate the role of the *Mapk8ip1* gene in β-cell function. Metabolic engineering using RNA-sequencing (RNA-seq) data confirmed higher expression levels of *MAPK8IP1* in human islets compared to other metabolic tissues. Additionally, comparable expression of MAPK8IP1 expression was detected in sorted human endocrine cells. However, β-cells exhibited higher expression of MAPK8IP1 than ductal and PSC cells. Notably, *MAPK8IP1* expression was reduced in diabetic islets, and the expression was positively correlated with insulin and the β-cell transcription factor *PDX1* and *MAFA.* Using the TIGER portal, we found that one genetic variant, “rs7115753,” in the proximity of MAPK8IP1, passes the genome-wide significance for the association with T2D. Expression silencing of *Mapk8ip1* by small interfering RNA (siRNA) in INS-1 cells reduced insulin secretion, glucose uptake rate, and reactive oxygen species (ROS) production. In contrast, insulin content, cell viability, and apoptosis without cytokines were unaffected. However, silencing of *Mapk8ip1* reduced cytokines-induced apoptosis and downregulated the expression of several pancreatic β-cell functional markers including, *Ins1*, *Ins2*, *Pdx1*, *MafA*, *Glut2*, *Gck*, *Insr*, *Vamp2*, *Syt5*, and *Cacna1a* at mRNA and/or protein levels. Finally, we reported that siRNA silencing of *Pdx1* resulted in the downregulation of MAPK8IP1 expression in INS-1 cells. In conclusion, our findings confirmed that MAPK8IP1 is an important component of pancreatic β-cell physiology and insulin secretion.

## 1. Introduction

Diabetes mellitus is characterized by insulin resistance, β-cell dysfunction, or both [1]. According to the International Diabetes Federation (http://www.idf.org, accessed on 1 February 2020), the number of people worldwide living with diabetes is 537 million. Type 2 diabetes (T2D) is the greatest prevalent type, representing almost 90% of all diabetic cases. Dysfunctional pancreatic β-cells are mediated by inflammatory cytokines and oxidative stress [2,3], which stimulate the c-Jun N-terminal kinase (JNK) signaling pathway [4]. Compelling evidence has demonstrated the involvement of the JNK signaling pathway in T2D [4,5,6]. The action of JNK necessitates the existence of a scaffold protein termed JNK interacting protein-1 (JIP-1) [7], which is abundantly expressed in the brain and pancreatic β-cells [8,9], as well as in several other tissues, such as muscle tissue, kidney tissue, and adipose tissue, but at lower levels [10,11]. The human and rat homologs of mouse JIP-1 protein were identified and named islet-brain1 (IB1) protein due to their abundant expression in insulin-producing β-cells and neurons [12]. JIP-1 or IB1 is also called mitogen-activated protein kinase 8 interacting protein-1 (MAPK8IP1). The gene symbol for human MAPK8IP1 is *MAPK8IP1*; the gene symbols for rat and mouse MAPK8IP1 are the same (Mapk8ip1).

In addition, MAPK8IP1 seems to be a multi-functional protein that may have other physiological functions. For example, MAPK8IP1 is localized in neurons [9] and has been implicated in c-Abl and Src tyrosine kinase signaling pathways [13,14]. Furthermore, MAPK8IP1 was also found to interact with the protein kinase Akt1 [15] and other proteins, such as IRS1 and IRS2 insulin receptors [11,16]. In addition, MAPK8IP1 acts as a regulatory protein for dynein-mediated and kinesin-mediated cargo transport along microtubules [17]. Thus, the JNK scaffold attributes of MAPK8IP1 demonstrate only one of several potential functions. 

The function of MAPK8IP1 in the evolution of diabetes is controversial. Earlier research proposed that MAPK8IP1 acts as a transactivator of the *GLUT2* gene [8] and identified it as a susceptibility gene for T2D, where a missense mutation in *MAPK8IP1* was found to segregate with T2D in humans [18]. However, this beneficial role of MAPK8IP1 has been challenged by several reports. For example, experiments of viable *Mapk8ip1*^−/−^ mice presented no evidence of a non-redundant role of MAPK8IP1 in pancreatic β-cells. Furthermore, *MAPK8IP1*-deficient mice (*Mapk8ip1*^−/−^) and Mapk8ip1-defective mice (*Mapk8ip1*^S59N/S59N^) did not exhibit a diabetic phenotype, in contrast to wild-type controls [16,19]. Moreover, Mapk8ip1-defective mice fed with high-fat diet (HFD) were protected against insulin resistance and obesity compared to HFD-fed WT mice [11,20]. On the other hand, while some reports have demonstrated the protective effect of MAPK8IP1 on insulin-secreting cells by regulating the activity of the JNK signaling pathway [21], others have shown further effects of MAPK8IP that were independent of JNK signaling [22]. 

To date, the function of MAPK8IP1 in pancreatic β-cell physiology and the pathogenesis of diabetes has not been entirely investigated. Therefore, this study aimed to examine the role of MAPK8IP1 on insulin secretion and β-cell function. This was accomplished by analysis of the RNA-sequencing data and monitoring the expression of *MAPK8IP1* in human pancreatic islets with/without diabetes. In addition, a series of functional experiments were carried out in INS-1 (832/13) cells to verify the impact of *Mapk8ip1* on the β-cell function.

## 2. Materials and Methods

### 2.1. Analysis of RNA-seq Data in Human Pancreatic Islets

The NCBI’s Gene Expression Omnibus (GEO) publicly available database was used to retrieve the RNA-seq expression data (https://www.ncbi.nlm.nih.gov/geo/query/acc.cgi?acc=GSE50398, accessed on 1 February 2020) [23]. As previously described, islets were previously processed [24] and RNA-sequencing was performed using Illumina’s TruSeq RNA Preparation Kit [23]. The output reads were aligned to the human reference genome (hg19) with STAR.17,18. Raw data were normalized using a trimmed mean of M-values and presented as fragments per kilobase of exon per million fragments mapped (FPKM) or transformed into log2 counts per million using the voom function (edgeR/limma R-packages). The expression data were obtained from 72 islet donors (European ancestry); of them, 51 were non-diabetic/normoglycemic donors (HbA1c < 6%; *n* = 51) and 21 were diabetic/hyperglycemic donors (6% ≤ HbA1c < 6.5%; *n* = 21).

### 2.2. Screening for T2D-Associated Genetic Variants in MAPK8IP1 

We searched a region spanning ±100 kb upstream and downstream of *MAPK8IP1* for T2D-genetic variants using the TIGER portal (http://tiger.bsc.es, accessed on 1 February 2020) [25]. As a result, three different GWAS meta-analysis datasets were explored: (i) the 70 K for the T2D project, consisting of 70,127 European ancestry subjects, including 13,857 T2D cases and 62,126 controls; (ii) the DIAGRAM 1000G, consisting of 159,208 European ancestry subjects, including 26,176 T2D cases and 132,532 controls; and (iii) the DIAGRAM Diamante T2D, consisting of 898,132 European ancestry subjects, including 74,124 T2D cases and 824,006 controls. Moreover, the T2D Knowledge Portal (T2DKP, containing 375 datasets and 377 traits) database (https://t2d.hugeamp.org, accessed on 1 February 2020) was also used for searching for T2D-genetic variants.

### 2.3. Culturing of INS-1 Cell Line and siRNA Transfection

Rat insulinoma INS-1 (832/13) were kindly provided by Dr. C. B. Newgaard, Duke University, USA [26]. As previously described, INS-1 (832/13) cells were cultured in RPMI-1640 medium supplemented with 10% fetal bovine serum [27]. For siRNA transfection, INS-1 cells were cultured in a 24-well plate (2 × 10^5^ cells/well) in an antibiotic-free RPMI 1640 medium. Upon reaching 60% confluency, cells were transfected with two sets of siRNA sequences against *Mapk8ip1* (s137914 and s137915) or *Pdx1* (s131652) or *MafA* (s172995) (Thermo Fisher Scientific, Waltham, MA, USA) or negative control siRNA, along with a mixture of Lipofectamine 3000 transfection reagent (Thermo Fisher Scientific, Waltham, MA, USA), as previously described [27]. The efficiency of siRNA transfection was monitored by a quantitative real-time reverse transcription polymerase chain reaction (qRT-PCR).

### 2.4. RNA Extraction and qRT-PCR 

The high-capacity cDNA Reverse Transcription Kit (Thermo Fisher Scientific, Waltham, MA, USA) was used for complementary DNA (cDNA) synthesis from the extracted RNA. Expression of key β-cell function genes in transfected and non-transfected cells was determined by qRT-PCR using TaqMan gene expression assays through the use of gene-specific primer probes for *Mapk8ip1* (Rn00587215_m1), *Ins1* (Rn02121433_g1), *Ins2* (Rn01774648_g1), *Glut2* (Rn00563565_m1), *Pdx1* (Rn00755591_m1), *Gck* (Rn00561265_m1), *Insr* (Rn00690703_ m1), and Rat *Hprt1* (Rn01527840_m1). SYBR green gene expression analysis was conducted using the primers (Table 1). The relative gene expression was done via the 2^−ΔΔCt^ method, and the qPCR reactions were performed at least three times. 

### 2.5. Insulin Secretion and Insulin Content Assay

Glucose-stimulated insulin secretion (GSIS) assay was conducted as previously described [27]. Briefly, for two hours, secretion assay buffer (SAB) containing 2.8 mM glucose was used to wash and pre-incubate silenced and control cells. The cells were then stimulated by SAB (1 mL) incubation, as previously reported [27]. Secreted insulin and insulin content were determined using a rat insulin enzyme-linked immunoassay (ELISA) kit (Mercodia, Uppsala, Sweden) and normalized to the total amount of protein. 

### 2.6. Glucose Uptake

A fluorescently labeled deoxyglucose analog, 2-[*N*-(7-nitrobenz-2-oxa-1,3-diazol-4-yl) amino]- 2-deoxy-glucose (2-NBDG) (Invitrogen #N13195, Waltham, MA, USA), was utilized as a probe for the detection of glucose uptake in cultured cells, as previously described [28]. Briefly, after 48 h transfection, cells were incubated with the 2-NBDG reagent (1 h). Cells were then trypsinized and analyzed by flow cytometry (BD FACS Aria III flow cytometer, San Jose, CA, USA) with excitation at 465 nm and emission at 540 nm.

### 2.7. Cell Viability Assay 

Cell viability assay was conducted using the 3-(4,5-dimethylthiazol-2-yl)-2,5-diphenyl tetrazolium bromide (MTT) colorimetric assay (Sigma–Aldrich, St. Louis, MO, USA) as previously described [28]. MTT solution (10 μL) was added to transfected cells 48 h post-transfection and incubated at 37 °C for 3–4 h. The absorbance was read as 570 nm using a microplate reader (crocodile mini work-station; Crocodile Control Software) and the percentage of cell viability was calculated.

### 2.8. Apoptosis Assay 

Transfected cells were cultured in RPMI medium in the presence and absence of a mixture of pro-apoptotic cytokines (IL-1β, 100 ng/mL; INFγ, 125 ng/mL and TNFα, 125 ng/mL) (Abcam, Cambridge, UK), as previously described [27]. Following 24 h incubation, the population of apoptotic cells was detected using the FITC Annexin-V kit (BD Biosciences, San Jose, CA, USA), as previously described [27]. Cells were analyzed using a BD FACS Aria III flow cytometer (Becton Dickinson Biosciences, San Jose, CA, USA).

### 2.9. Reactive Oxygen Species (ROS) Measurements 

Intracellular ROS was detected using a ROS-Glo H_2_O_2_ assay kit (Cat #G8820, Promega, Madison, WI, USA). In brief, 48 h post-transfection, INS-1 cells were incubated with H_2_O_2_ substrate (3 h) at 37 °C. After incubation, ROS-Glo detection reagent (100 μL) was added to all wells and cells were incubated for another 20 min (RT). The relative luminescence was then detected using a plate reader [28].

### 2.10. Western Blot Analysis 

Protein extractions were carried out using ice-cold NP-40 lysis containing protease inhibitor cocktail (Thermo Fisher Scientific, Waltham, MA, USA) [28]. The standard Bradford method (Bio-Rad, Hercules, CA, USA) measured the protein concentration. Western blot analyses were conducted, as previously described [28], using the following primary antibodies: MAPK8IP1 (anti-rabbit; 1:1000, #Ab24449, Abcam), Pro/Insulin (anti-mouse; 1:1000; #8138s, Cell Signaling Technology, Danvers, MA, USA), INSRβ (Anti-rabbit; 1:1000; #23413, Cell Signaling Technology, USA), PDX1 (anti-rabbit; 1:1000, #ab47267, Abcam, Cambridge, UK), MAFA (anti-rabbit; 1:1000, #ab264418, Abcam, Cambridge, UK), GLUT2 (anti-rabbit; 1:1000, #A12307, Abclonal), NEUROD1 (anti-rabbit; 1:1000, #ab213725, Abcam, Cambridge, UK), GCK (anti-rabbit; 1:100; #ab37796, Abcam, Cambridge, UK), VAMP2 (anti-rabbit; 1:1000, #13508, Cell Signaling Technology, USA), SNAP25 (anti-mouse, 1:1000, #MA5-17609, Invitrogen), JNK (anti-rabbit; 1:1000, #A48567, Abclonal), pJNK (anti-rabbit; 1:1000, #AP0631, Abclonal) or β-actin (anti-mouse, 1:1000, #A5441, Sigma–Aldrich, Taufkirchen, Germany). Horseradish peroxidase-linked anti-rabbit (#7074S, Cell Signaling Technology, USA) or anti-mouse (#7076S, Cell Signaling Technology, USA) antibodies were used as secondary antibodies. Quantification of the protein bands using the Bio-Rad Image Lab software (ChemiDocTM Touch Gel Western Blot Imaging System; Bio-Rad, Hercules, CA, USA) and the β-actin was employed as an internal control. 

### 2.11. Statistical Analysis 

Unless otherwise stated, the data are presented as means ± SEM (standard error of the mean) or ± SD (standard deviation) from at least three independent experiments, as indicated in each experiment in figure legends. The median MAPK8IP1 expression between the two groups in human islets was compared using the non-parametric Mann–Whitney test. The correlation between the expression of MAPK8IP1 and other genes/variables was determined using the non-parametric Spearman test. For the qPCR, Western blotting, cell viability, and insulin secretion experiments, the differences between negative control and MAPK8IP1-silenced cells were compared using the Student’s T-test. All the statistical analyses were conducted using GraphPad Prism (version 8.0.0 for Windows, GraphPad Software, San Diego, CA, USA, www.graphpad.com, accessed on 1 October 2022). *p*-values < 0.05 were considered significant. * indicates results with *p* < 0.05, ** *p* < 0.01, *** *p* < 0.001.

## 3. Results

### 3.1. Expression Profile of MAPK8IP1 in Diabetic and Non-Diabetic Human Pancreatic Islets

**Figure 1:**
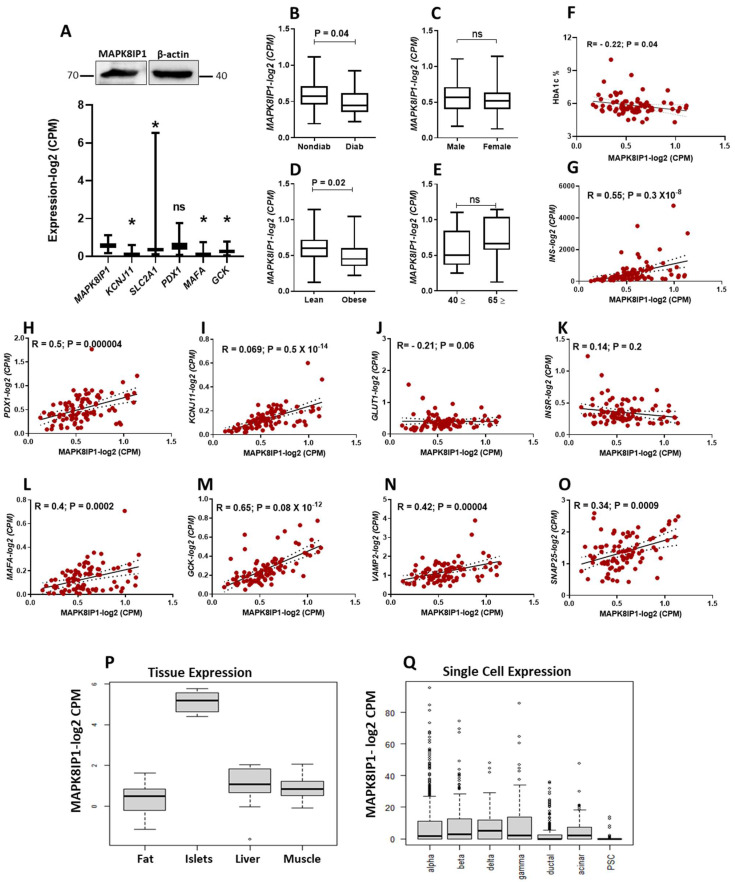
Expression profile of *MAPK8IP1* in human pancreatic islets. (**A**) RNA-seq expression of *MAPK8IP1*, *KCNJ11*, *SLC2A1*, *PDX1*, *MAFA* and *GCK* in non-diabetic human islets (*n* = 57) (lower panel). Western blot expression analysis of MAPK8IP1 in human islets obtained from non-diabetic donors (*n* = 1; obtained from Prodo Lab, Aliso Viejo, CA, USA) (upper panel). (**B**–**E**) RNA-seq differential expression analysis of *MAPK8IP1* in human islets obtained from diabetic/hyperglycemic donors (*n* = 21) vs. nondiabetic/normoglycemic donors (*n* = 51) (**B**), male donors (*n* = 54) vs. female donors (*n* = 36) (**C**), lean donors (BMI < 25; *n* = 39) vs. obese donors (BMI > 29; *n* = 20) (**D**), old donors (≥65; *n* = 9) vs. young donors (≤40; *n* = 14) (**E**). (**F**–**O**) Co-expression correlations of *MAPK8IP1* with HbA1c (**F**), *INS* (**G**), *PDX1* (**H**), *KCNJ11* (**I**), *GLUT1* (**J**), *INSR* (**K**), *MAFA* (**L**), *GCK* (**M**), *VAMP2* (*n*), and *SNAP25* (**O**) using RNA-seq expression data from human pancreatic islets. R and *p* values are indicated in the respective graphs. (**P**) *MAPK8IP1* expression in human fat tissue (*n* = 12), pancreatic islets (*n* = 12), liver (*n* = 12) and skeletal muscle tissues (*n* = 12) obtained from the same donors using IGV data. (**Q**) *MAPK8IP1* expression in sorted pancreatic cells, ductal, acinar, or PSC was obtained using IGV data. R: correlation coefficient; *p*: *p*-value. * *p* < 0.05; ns, not significant. Bars above histograms represent SD of the mean values. We assessed the expression using the nonparametric Mann–Whitney test for figures (**A**–**E**). For figure (**F**–**O**), we evaluated the correlations using the nonparametric Spearman’s test.

To understand whether MAPK8IP1 has a potential role in the function of pancreatic β-cells, we first explored its mRNA expression in healthy human pancreatic islets (*n* = 57) using published RNA-seq gene expression data (https://www.ncbi.nlm.nih.gov/geo/query/acc.cgi?acc=GSE50398, accessed on 1 February 2020) [23]. As shown in the lower panel of Figure 1A, expression analysis revealed that *MAPK8IP1* is expressed in healthy human islets. Remarkably, the expression of *MAPK8IP1* was higher than some functional β-cell genes, including *KCNJ11* or *SLC2A1* (*GLUT1*), *MAFA*, and *GCK* genes (Figure 1A, lower panel). *PDX1* exhibited an expression profile comparable to that of *MAPK8IP1* (*p* > 0.05). Moreover, we confirmed the expression of MAPK8IP1 in human islets at protein levels from non-diabetic islets (*n* = 1, obtained from Prodo Lab, Aliso Viejo, CA, USA) (Figure 1A, upper panel). Exploration of the effect of diabetes/hyperglycemia status on the mRNA expression of MAPK8IP1 showed a significant reduction in the case of diabetic/hyperglycemic islets (HbA1C ≥ 6%; *n* = 21) compared to nondiabetic/normoglycemic islets (HbA1c < 6%; *n* = 50) (*p* = 0.04) (Figure 1B). We noticed a significant decrease in the expression of *MAPK8IP1* in obese donors (BMI > 29; *n* = 20) compared to lean donors (BMI < 25; *n* = 39) (*p* < 0.05) (Figure 1D). However, no differences in mRNA expression of MAPK8IP1 were observed based on gender (male, *n* = 54 versus female, *n* = 36) or age (old donors ≥ 65, *n* = 9 versus young ≤ 40, *n* = 14) (Figure 1C,E). The expression of *MAPK8IP1*, however, correlated negatively with HbA1c levels (Figure 1F). Next, we correlated the expression of *MAPK8IP1* with the expression of pancreatic β-cells’ key functional genes. *MAPK8IP1* expression correlated positively (*p* < 0.05) with that of *INS*, *KCNJ11*, *PDX1*, *MAFA*, *GCK*, *VAMP2*, and *SNAP25* and negatively with that of *GLUT1* (Figure 1G–O). *INSR* expression showed a non-significant correlation with MAPK8IP1 (Figure 1K). Furthermore, the Islet Gene View (IGV) web tool (https://mae.crc.med.lu.se/IsletGeneView/, accessed on 1 October 2022) was used to assess *MAPK8IP1* expression in different metabolic tissues [29]. As shown in Figure 1P, MAPK8IP1 showed high expression in human islets compared to other metabolic tissues. Using IGV data, we also highlighted the expression of *MAPK8IP1* in sorted pancreatic endocrine cells. Pancreatic β-cells showed expression levels of *MAPK8IP1* similar to those of other endocrine cells but higher than those of ductal and PSC cells. Among sorted endocrine cells, *MAPK8IP1* expression in delta cells showed the highest expression level (Figure 1Q). Finally, based on GTEx data (www.gtexportal.org, accessed on 1 October 2022). GTEx Analysis Release V8; dbGaP Accession phs000424 v8, p2), distinct levels of *MAPK8IP1* expression were also detected across diverse human tissue, with neurons and brain tissue showing higher *MAPK8IP1* expression than other tissues (Figure S1).

### 3.2. Exploration of T2D Genetic Variants in MAPK8IP1

Using the recently developed tool “TIGER portal” (http://tiger.bsc.es, accessed on 1 February 2022) [25], we examined whether *MAPK8IP1* contains any T2D-associated genetic variants (single nucleotide polymorphism, SNP). Three different data sets were searched (70K for T2D project, DIAGRAM Diamante, and DIAGRAM 1000 G). The top T2D-associated genetic variants of each dataset are listed in Table 2. We found that rs7115753 passes the significance threshold at the genome-wide significance threshold (*p* < 5.0 × 10^−8^). Moreover, we explored GWAS datasets for the association by utilizing the T2D Knowledge Portal (T2DKP, containing 375 datasets and 377 traits) database of rs7115753, rs11038677, and rs553011963 with T2D and other related traits. The rs7115753 showed a significant association with fasting glucose adjusted to BMI (*p* = 9.76 × 10^−51^), fasting glucose (*p* = 2.75 × 10^−13^), T2D adjusted with BMI (*p* = 1.37 × 10^−7^), and T2D (*p* = 1.62 × 10^−7^). Similarly, rs11038677 showed a significant association with fasting glucose adjusted to BMI (*p* = 2.45 × 10^−24^), fasting glucose (*p* = 2.37 × 10^−8^), and T2D adjusted with BMI (*p* = 3.59 × 10^−7^) (Table 3).

### 3.3. Silencing of Mapk8ip1 in INS-1 (832/13) Cells Affects Insulin Secretion and Glucose Uptake

**Figure 2:**
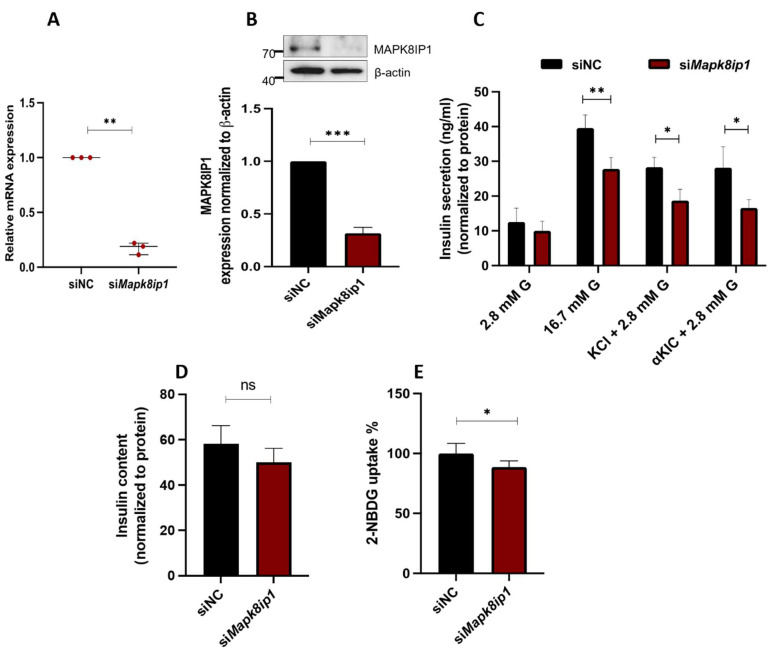
Silencing of *Mapk8ip1* diminishes insulin secretion in INS-1 cells. Cells were transfected with siRNA against *Mapk8ip1* (*siMapk8ip1*) or the siRNA negative control (siNC). (**A**) The silencing efficiency of *Mapk8ip1* was measured by qRT-PCR 48 h post-transfection. Data were obtained from three independent experiments. (**B**) Western blot expression analysis of MAPK8IP1 and β-actin (endogenous control) in INS-1 cells transfected with siRNA against *Mapk8ip1* or the negative control (upper panel). Fold change in the intensity of the Western blot band of MAPK8IP1 protein relative to the endogenous control β-actin in *Mapk8ip1*-silenced cells or negative control cells (lower panel). Data were obtained from three independent experiments. (**C**) Normalized stimulated insulin secretion in response to 2.8 mM glucose (2.8 mM G), 16.7 mM glucose (16.7 mM G), 35 mM KCl, or 10 mM α-KIC in the presence of 2.8 mM glucose in *Mapk8ip1*-silenced cells or negative control cells for one static hour of incubation. Data were obtained from three independent experiments. (**D**) Insulin content measurements normalized to protein content in *Mapk8ip1*-silenced cells compared to negative control cells. Data were obtained from three independent experiments. (**E**) Evaluation of glucose uptake efficiency in *Mapk8ip1*-silenced cells compared to control cells. Data were obtained from three independent experiments. * *p* < 0.05, ** *p* < 0.01, *** *p* < 0.001 and ns; not significant. Bars represent mean ± SD. Statistical analyses were performed using Student *t*-tests.

To understand the impact of *Mapk8ip1* on the function of pancreatic β-cells, we used a pool of siRNA sequences to ablate its expressions in INS-1 (832/13) cells. Expression analysis of *Mapk8ip1* 48 h post-transfection, assessed by qPCR, demonstrated a significantly lower expression in silenced cells (~82%; *p* < 0.05) than in negative control cells (Figure 2A). This was further confirmed by Western blot analysis (~60%; *p* < 0.05) (Figure 2B). To evaluate the consequences of *Mapk8ip1* silencing on insulin secretion, transfected cells were incubated (1 h) with 2.8 mM glucose (basal level) or 16.7 mM glucose (stimulation level). As shown in Figure 2C, a significant reduction in GSIS at 16.7 mM glucose (~30%; *p* < 0.05) was observed in *Mapk8ip1*-silenced cells compared to control cells. However, no change was noticed at the basal level (2.8 mM glucose). 

Moreover, the effect of *Mapk8ip1* silencing on the insulin exocytosis machinery was evaluated by stimulating the transfected cells with 35 mM potassium chloride (a depolarizing agent) or 10 mM alpha ketoisocaproic acid (an agent that stimulates mitochondrial metabolism and ATP synthesis) for 1 h. As illustrated in Figure 2C, significant reductions in KCl-stimulated (~33%; *p* < 0.05) and α-KIC-stimulated (~40%; *p* < 0.05) insulin secretion were observed, compared to the negative control. On the other hand, no significant change was observed upon measurement of insulin content in *Mapk8ip1*-silenced cells compared to the control cells (Figure 2D). Finally, silencing of *Mapk8ip1* exhibited a significant reduction (~15%; *p* < 0.05) in the level of glucose uptake, as determined by 2-NBDG assay, compared to control cells (Figure 2E).

### 3.4. Effect of Mapk8ip1 Silencing on Cell Viability, Apoptosis, and ROS in INS-1 (832/13) Cells

**Figure 3:**
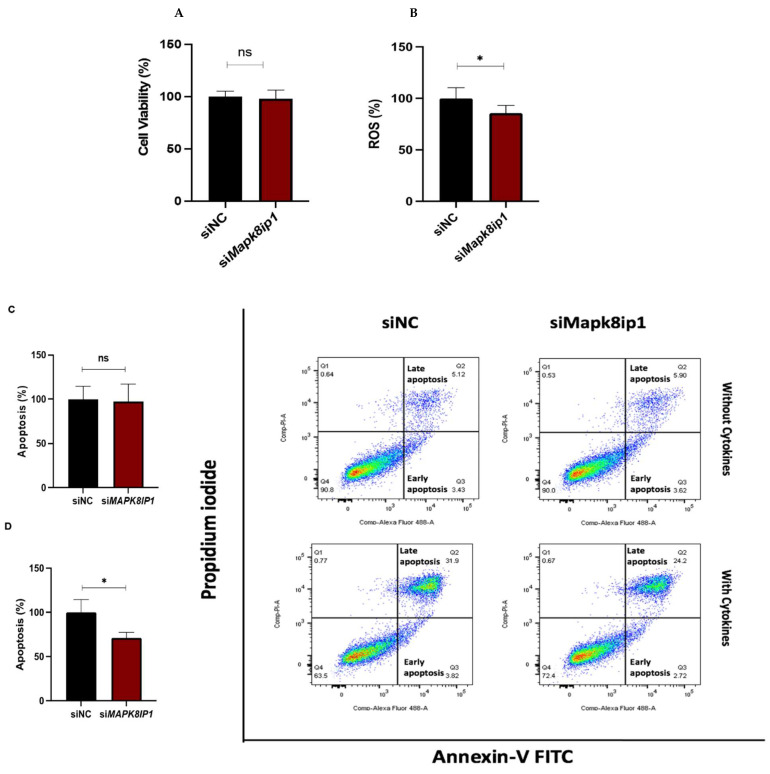
Influence of *Mapk8ip1*-silencing on cell viability, apoptosis, and ROS production in INS-1 cells. Cells were transfected with siRNA against *Mapk8ip1* (si*Mapk8ip1*) or the siRNA negative control (siNC). (**A**) Percentage of cell viability determined by MTT assay in *Mapk8ip1*-silenced cells compared to control cells. Data were obtained from three independent experiments. (**B**) Detection of intracellular ROS levels in *Mapk8ip1*-silenced INS-1 cells compared to control cells. Data were obtained from three independent experiments. The ROS level in the control cells was set at 100%. Analysis of apoptosis level in *Mapk8ip1*-silenced INS-1 cells or negative control in the absence (**C**) or presence (**D**) of cytokine stimulation as analyzed by flow cytometry. Data were obtained from three independent experiments. * *p* < 0.05, ns, not significant. Bars above histograms represent SD of the mean values. Statistical analyses were performed using Student *t*-tests.

Herein, we studied the effect of *Mapk8ip1* silencing on cell viability, apoptosis, and ROS production. As illustrated in Figure 3A, the assessment of cell viability using MTT assay showed no differences between *Mapk8ip1*-silenced cells and the negative control cells. However, we noticed a significant reduction in intracellular ROS production (14%, *p* < 0.05) in *Mapk8ip1*-silenced cells compared to that in control cells (Figure 3B). On the other hand, the percentage of apoptotic cells assessed by Annexin-V staining in the absence of pro-apoptotic cytokines showed no difference in *Mapk8ip1*-silenced cells compared to control cells (Figure 3C). However, *Mapk8ip1*-silenced cells, in the presence of a mixture of pro-apoptotic cytokines (IL-1β, TNFα, and INFγ) for 24 h, showed a significant reduction (~29%) in cytokine-induced apoptosis compared to negative control cells (*p* < 0.05) (Figure 3D).

### 3.5. Mapk8ip1 Silencing Modifies the Expression of β-Cell Function-Related Genes

**Figure 4:**
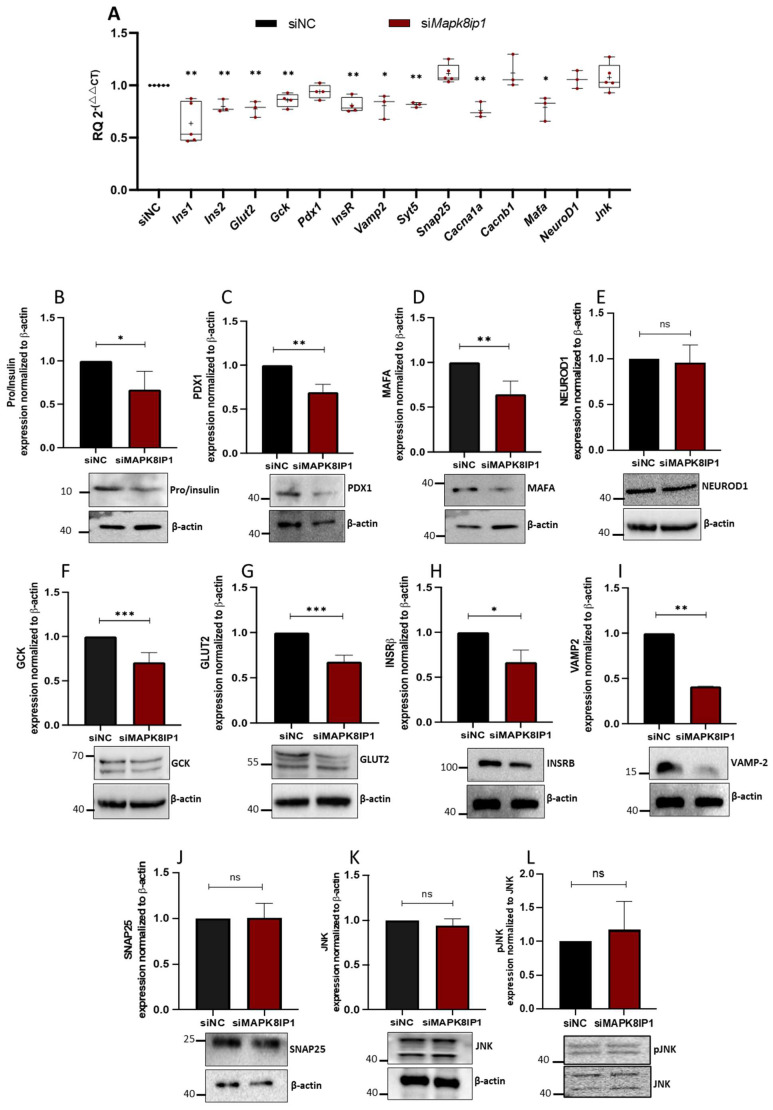
Impact of *Mapk8ip1* silencing on the expression of β-cell function genes. Total mRNA/protein was extracted from *Mapk8ip1*-silenced cells (si*Mapk8ip1*) or siRNA negative control cells (siNC) after 48 h of transfection and subjected to qRT-PCR or Western blot analysis. (**A**) qPCR expression analysis of *Ins1*, *Ins2*, *Glut2*, *Gck*, *Pdx1*, *Insr*, *Vamp2*, *Syt5*, *Snap25*, *Cacna1a*, *Cacnb1*, *MafA*, *NeuroD1*, and *Jnk* in *Mapk8ip1*-silenced cells compared to those in control cells. Data were obtained from at least three independent experiments.Western blot analysis of Pro/insulin (**B**), PDX1 (**C**), MAFA (**D**), NEUROD1 (**E**), GCK (**F**), GLUT2 (**G**), INSRβ (**H**), VAMP2 (**I**), SNAP25 (**J**), JNK (**K**) and pJNK (**L**) relative to the endogenous control protein β-actin (pJNK levels shown relative to JNK) in *Mapk8ip1*-silenced cells and control cells (lower panels). Corresponding fold changes in the intensities of the Western blot bands are shown above each blot. Data were obtained from three independent experiments (see Supplementary Figures S2–S4). * *p* < 0.05, ** *p* < 0.01, *** *p* < 0.001, ns; not significant. Bars above histograms represent SD of the mean values. Statistical analyses were performed using Student *t*-tests.

We next investigated the impact of *Mapk8ip1* silencing on the expression of functional genes in β-cells at transcriptional and translational levels. As illustrated in Figure 4A, transient silencing of *Mapk8ip1* in INS-1 cells was linked with a significant reduction (*p* < 0.05) in mRNA levels of genes involved in insulin biosynthesis (*Ins1*, *Ins2*, *MafA*), glucose sensing genes (*Glut2*, *Gck*), and insulin exocytotic regulatory genes (*Cacna1a*, *Vamp2*, *Syt5*) compared to those in control cells (Figure 4A). Expression of *Pdx1*, *NeuroD1*, *Cacnb1*, *Snap25*, and *Jnk* was not significantly affected. In addition, expression of *Insr,* which is involved in insulin signaling, was decreased (*p* < 0.05) in *Mapk8ip1*-silenced cells at the mRNA level (Figure 4A). At protein levels, a significant downregulation (*p* < 0.05) was observed in pro/insulin (~33%), PDX1 (~30%), MAFA (~35%), GCK (~29%), GLUT2 (~30%), INSRβ (~33%), and VAMP2 (~58%) in *Mapk8ip1*-silenced cells versus control cells (Figure 4B–L). In contrast, protein expression of NEUROD1, SNAP25, JNK, and phosphorylated JNK (pJNK) was not affected (Figure 4B–L). The replicas of the full-length Western blot expressions after *Mapk8ip1* silencing are displayed in Figures S2–S4.

### 3.6. PDX1 Is Required for Proper MAPK8IP1 Expression in β-Cells

**Figure 5:**
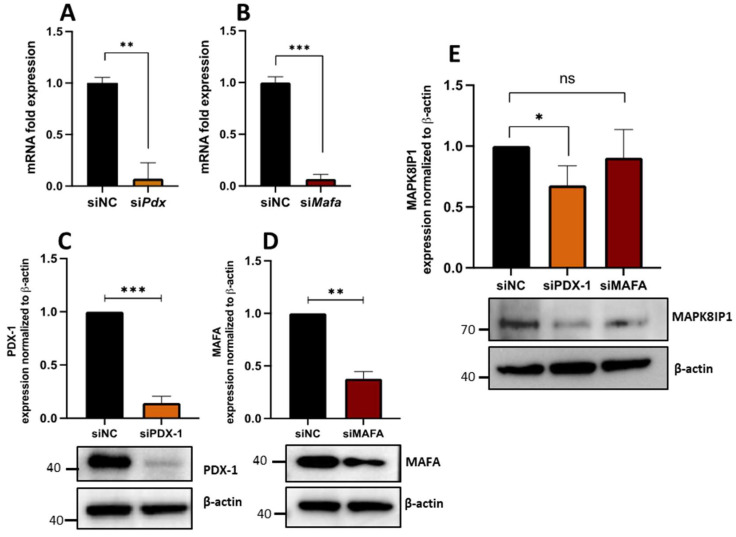
Impact of *Pdx1* and *MafA* silencing on the expression of MAPK8IP1 in INS-1 cells. Cells were transfected with siRNA against *Pdx1* (*siPdx1*) or *MafA* (si*MafA*), or the siRNA negative control (siNC). (**A**) Silencing efficiency of *Pdx1* in INS1 cells 48 h post-transfection as measured by qRT-PCR. (**B**) Silencing efficiency of *MafA* in INS1 cells 48 h post-transfection as measured by qRT-PCR qPCR. (**C**–**E**) Western blot analysis of PDX1 (**C**), MAFA (**D**) and MAPK8IP1 (**E**) in *Pdx1* or *MafA*-silenced cells and control cells relative to the endogenous control protein β-actin (lower panels). Corresponding fold changes in the intensities of the Western blot bands are shown above each blot. Data were obtained from three independent experiments (see Supplementary Figures S5–S7). * *p* < 0.05, ** *p* < 0.01, *** *p* < 0.001, ns; not significant. Bars above histograms represent SD of the mean values. Statistical analyses were performed using Student *t*-tests.

The transcription factors PDX1 and MAFA are critical regulators for β-cell identity and function [30,31,32,33]. To determine if the loss of *Pdx1* or *MafA* in β-cells affects *Mapk8ip1* expression in INS-1 cells, we silenced the expression of both genes (*Pdx1* and *MafA*), followed by measurement of MAPK8IP1 expression levels. As shown in Figure 5A,B, siRNA silencing of *Pdx1* or *MafA* significantly reduced the expression of each gene (*p* < 0.001), as determined by qPCR analysis. Knockdown was further validated at protein levels (*p* < 0.001) (Figure 5C,D). Notably, a significant downregulation of MAPK8IP1 expression was observed in *Pdx1*-silenced cells (*p* < 0.05) compared to control cells (Figure 5E). However, MAPK8IP1 expression in *MafA*-silenced cells was not affected (Figure 5E). The replicas of the full-length Western blot expression after si*MafA* and si*Pdx1* silencing are shown in Figures S5–S7.

## 4. Discussion

Although the expression of *MAPK8IP1* and its function as a transactivator of the glucose transporter gene (*GLUT2*) have been demonstrated in pancreatic β-cells [8], its role in the physiology of β-cells is not fully clear and is subject to great controversy. This work presents an elaborative study that used a combined analysis of publicly available data and new functional studies to investigate different perspectives of the role of MAPK8IP1 in the physiology of β-cells. In the present study, we demonstrated that *MAPK8IP1* is present in human islets and that expression was reduced in diabetic or obese donors. Furthermore, expression of *MAPK8IP1* was correlated with insulin expression and the key transcription factors in β-cells. In the search for T2D-associated genetic variants in *MAPK8IP1* using the TIGER portal, we found that rs7115753 passes the genome-wide significance threshold for association with T2D. Moreover, expression silencing of *Mapk8ip1* in INS-1 cells reduced insulin secretion, ROS, and glucose uptake levels.

In contrast, insulin content, cell viability, and apoptosis in the absence of added cytokines (IL-1β, INFγ, and TNFα) were not affected. We also demonstrated that *Mapk8ip1* silencing reduced cytokines-induced apoptosis and altered the expression of several pancreatic β-cell functional genes at the mRNA and protein levels. Taken together, our findings suggest that *MAPK8IP1* is an important component of pancreatic β-cell physiology and insulin secretion.

Expression mapping of *MAPK8IP1* in human islets showed high expression relative to other restricted β-cell functional genes such as *KCNJ11* and *MAFA* (Figure 1A). Furthermore, *MAPK8IP1* expression was revealed to be lower in other metabolic tissues, such as liver tissues, adipose tissues, or muscle tissues, compared to human islets. We also highlighted the expression of *MAPK8IP1* in sorted pancreatic endocrine cells, where pancreatic β-cells showed expression levels of *MAPK8IP1* that were similar to those of other endocrine cells but higher than those of ductal and PSC cells. These data are in line with another RNA-seq study that reported that the expression of *MAPK8IP1* in human β-cells was 5.4 (normalized expression values), 5.2 in α-cells, and 4.9 in exocrine cells [34]. Thus, these data suggest that *MAPK8IP1* is expressed in both endocrine and exocrine pancreatic cells.

On the other hand, our findings showed that *MAPK8IP1* is reduced in diabetic/hyperglycemic islets. This raises the question of whether the reduction of *MAPK8IP1* is involved in the pathogenies of T2D or just a consequence of hyperglycemia exposure (glucotoxicity). A study by Ottosson-Laakso et al. found that short-term incubation of human islets in high glucose concentrations showed no change in the expression of *MAPK8IP1* [35], which rules out the impact of short-term glucotoxicity. However, the effect of long-term exposure needs further investigation. Although it is still premature to claim that the observed reduction in *MAPK8IP1* in diabetic islets is involved in the pathophysiology of diabetes, the negative correlation of *MAPK8IP1* expression with HbA1c levels and positive correlation with β-cell functional genes indicates the important role of *MAPK8IP1* in the physiology of β-cell. A third possible explanation behind the reduction of *MAPK8IP1* in diabetic islets could be a compensatory mechanism that regulates the expression.

Among the top genetic variants in the proximity of *MAPK8IP1,* we found that rs7115753 passed the genome-wide significance for the association with T2D. The rs7115753 is an intronic SNP located at chr11:45890462 (hg38/Human) and may not directly influence MAPK8IP1 expression. However, it might exist in linkage disequilibrium with other functional SNPs and play a role in disease risk. Moreover, intronic SNPs are also known to influence genes that are not in close proximity to them. For instance, an obesity-related SNP found in the intron of the FTO gene was found to influence the process of adipocyte browning by regulating the activity of IRX3 and IRX5, rather than directly impacting FTO itself [36,37]. Thus, further investigation is warranted to address the putative effect of the rs7115753 on the function of MAPK8IP1 or in cis (within 1 Mb of the SNP) and in trans (further than 1 Mb away or on a different chromosome).

Furthermore, rs7115753 and rs11038677 were significantly associated with fasting glucose adjusted to BMI, fasting glucose, and T2D adjusted to BMI in MAGIC 2021 glycemic traits GWAS. This interesting finding may pave the way to explore whether these genetic variants are associated with other traits in different populations.

The role of MAPK8IP1 in the development of diabetes is disputable. Earlier studies identified MAPK8IP1 as a susceptibility gene for T2D, where a missense mutation in MAPK8IP1 was found to segregate with T2D in humans [18]. Furthermore, the gene was proposed to act as a transactivator of the GLUT2 gene [8]. This was disputed in several reports that revealed a redundant role of MAPK8IP1 on β-cells and insulin sensitivity, where the absence of a detected diabetic phenotype was demonstrated in *Mapk8ip1*-deficient mice [19]. Moreover, Kant et al. [20] and Jaeschke et al. [11] demonstrated that HFD-fed *Mapk8ip1*-defective mice were protected against obesity and insulin resistance, compared to HFD-fed WT mice.

On the other hand, some reports have demonstrated a protective effect of MAPK8IP1 on insulin-secreting cells by regulating the activity of the JNK signaling pathway [21]. Alternatively, other reports have revealed pro- and anti-apoptotic effects of MAPK8IP1, independent of JNK signaling [22]. To date, the function of MAPK8IP1 in pancreatic β-cell physiology and the pathogenesis of diabetes remains elusive.

Previous studies reported an anti-apoptotic function of MAPK8IP1 and attributed this to its role in regulating the JNK signaling pathway [21,38]. Our results demonstrate that the silencing of *Mapk8ip1* had no effect on apoptosis without cytokines or on basal pJNK levels but decreased cytokine-induced apoptosis. In line with our data, *Mapk8ip1* knockdown was found to reduce stress-induced apoptosis in different settings [19,39]. Moreover, Ling et al. demonstrated that MAPK8IP1 protein possesses pro-apoptotic and anti-apoptotic properties independently of the effects on the JNK signaling cascade [22]. Three JNK genes have been recognized, all of which were detected in β-cells [40]. While JNK2 seems pro-apoptotic, JNK1 and JNK3 are anti-apoptotic [40,41]. The balance between the expression of the different JNK isoforms in concluding total JNK activity may be a critical determinant of β-cell survival. Therefore, additional investigations are needed to assess whether MAPK8IP1 could regulate each of the three JNK isoforms and the effect of such regulation on β-cell survival. Nevertheless, differences in the culture conditions, the concentrations used, the transfection protocol, the experimental setup, and the choice of assay used to measure cell death cannot be ruled out as possible explanations for the discrepancies between our data and the results from previous studies.

ROS imbalance plays a central role in β cell dysfunction and the pathogenesis of diabetes [42]. However, the impact of MAPK8IP1 on the generation of ROS has not been previously investigated. Our results showed a reduction of ROS via *Mapk8ip1* silencing. This finding was not surprising, as it has been shown that diminished levels of ROS signaling may result in reduced insulin secretion [43].

Few studies have investigated the link of MAPK8IP1 with insulin secretion [8,18]; however, its effect on insulin content and glucose uptake has not been elucidated. Our study illustrates that expression silencing of *Mapk8ip1* in INS-1 cells was associated with impaired insulin secretion and decreased glucose uptake efficiency but had no effect on insulin content. The reduced glucose uptake is speculated to contribute to the impaired GSIS observed, as glucose uptake via glucose transporters is one of the key events leading to insulin secretion.

To achieve greater insight into how *Mapk8ip1* reduced insulin secretion and the cause of reduced glucose uptake, we studied the expression of several key β-pancreatic cell genes that are mainly involved in glucose sensing, insulin biosynthesis, and insulin release. Intriguingly, *Mapk8ip1* silencing altered the expression of several functional genes in β-cells. Most studies focused on the impact of *Mapk8ip1* on *Ins* and *Glut2* gene expressions, while its effect on other crucial key β-cell functional genes has not been studied. It was reported that MAPK8IP1 is a candidate for T2D and is required for insulin and GLUT2 expression [18]. Data presented in this study support the notion that MAPK8IP1 is involved in regulating insulin secretion [8,18] and undoubtedly unravels the role of *Mapk8ip1* silencing on the exocytosis machinery and mitochondrial metabolism. Our results showed that *Mapk8ip1*-silenced cells exhibited a downregulation of genes involved in insulin biosynthesis, *Ins1* and *Ins2,* along with the transcription factor genes *Pdx1* and *MafA*. The *Pdx1* gene controls the maturation of β cell function and insulin secretion by regulating insulin gene expression [44]. The *MafA* gene regulates the expression of insulin [45] and is necessary for the homeostasis of mature beta cells [46]. Thus, we can speculate that knockdown of *Mapk8ip1* reduced the *Pdx1* and *MafA* expression pattern, which resulted in the observed reduced insulin secretion and β-cell dysfunction. *Glut2* and *Gck* are both crucial elements in glucose-sensing machinery and uptake. Typically, defects in glucose-sensing machinery are associated with impaired insulin secretion and hyperglycemia [47,48]. In the current study, GLUT2 and GCK expression was also downregulated in *Mapk8ip1*-silenced cells at both mRNA and protein levels. These results are in contrast with previously published reports that suggested that GLUT2 expression in *Mapk8ip1*-silenced cells was not significantly different from that in control cells [22]. It is worth noting that the reduced expression of GLUT2, the principal glucose transporter in beta cells, was associated with decreased glucose uptake, suggesting the contribution of GLUT2 to reduced glucose uptake and GSIS observed in our study.

Additionally, the downregulation of Insr at mRNA and protein levels in *Mapk8ip1*-silenced cells, observed in our study, might contribute to the observed insulin secretion defect; studies have demonstrated that the ablation of *Insr* in beta cells can impair insulin secretion [49]. Finally, our findings show that the expression levels of genes involved in insulin exocytosis (*Syt5*, *Vamp2*, and *Cacna1a*) were also reduced in *Mapk8ip1*-silenced cells and associated with a reduction in KCl-stimulated insulin secretion. *Vamp2* is involved in the docking of insulin secretory vesicles with the target membrane [50]; *Syt5* mediates Ca^2+^-dependent exocytosis of insulin [51], while *Cacna1a* plays an important role in insulin release, both as a source of Ca^2+^ required for excitation–secretion coupling and as a scaffold for the release machinery [52]. Thus, the impairment of these insulin exocytosis genes may be another contributing factor to the reduced GSIS observed in *Mapk8ip1*-silenced cells.

Importantly, we noticed that *Mapk8ip1* levels were reduced, at both transcription and translation levels, upon the silencing of *Pdx1* in INS-1 cells. These data suggest that *Mapk8ip1* expression is dependent on *Pdx1* transcriptional activity. This is an interesting finding, as previous reports documented that a large fraction of diabetic patients with mutant *Mapk8ip1* allele were also carriers of a *Pdx1* mutation, which was previously associated with a late-onset form of diabetes [18]. Furthermore, the finding that *Mapk8ip1* silencing impaired mRNA and/or protein expression of *Pdx1* and reduced insulin secretion represents a β-cell dedifferentiation condition. This might suggest that the reduced *Mapk8ip1* expression in diabetic islets may be direct or through loss of *Pdx1*.

It is worth noting that this study has some limitations. In this study, we utilized INS-1 cells as the validation in vitro model. However, certain gene expression variations due to species-specificity may exist between the rat INS-1 cells and human β-cells. This might imply functional differences across the different species. Hence, it is of great importance to further validate the findings using human islets in future studies.

## 5. Conclusions

In summary, our data proposed that *MAPK8IP1* is involved in the physiology of pancreatic beta cells. *Mapk8ip1* silencing reduced cytokines-induced apoptosis and downregulated the expression of several pancreatic β-cell functional genes, including *Ins1*, *Ins2*, *MafA*, *Glut2*, *Gck*, *Insr*, *Vamp2*, *Syt5*, and *Cacna1a*. Furthermore, siRNA silencing of *Pdx1* resulted in downregulated *Mapk8ip1* expression in INS-1 cells. Further work is required to translate the findings to human islets and to determine whether MAPK8IP1 may represent a possible target for therapeutic interventions or be used as a biomarker for β-cell dysfunction.

## Figures and Tables

**Table 1:** SYBR green primer sequence.

Gene/Symbol	Accession Numbers	Forward Primers (5′-3′)	Reverse Primers (5′-3′)
*MafA*	NM_001399773.1	GAGGAGGAGCGCAAGATCGG	AGCAAAAGTTTCGTGCTGTCAA
*NeuroD1*	NM_019218.3	CCCTAACTGATTGCACCAGC	TGCAGGGTAGTGCATGGTAA
*Syt5*	NM_019350.2	CACCTGACCCCAGATCCTTT	GAGTGGTACTGGAAGTCGGA
*Snap25*	NM_001270576.1 NM_001270575.1 NM_030991.3	GGCGTTTGCTGAATGACAAC	CAGAGCCTGACACCCTAAGA
*Cacna1a*	NM_012918.5	CTAGCCCTGCCAAGATCGG	ACGATAAGGCTGTTCTCGG
*Cacnb1*	NM_017346.2	CTTTACCCCAGCAACCACCC	GTCCACACACGAGTCTCCTG
*Vamp2*	NM_012663.2	TGGTGGACATCATGAGGGTG	GCTTGGCTGCACTTGTTTCA
*Jnk*	NM_053829.2	TCCAGTTCTCGTACCCGCTA	AGCATGGCGTGACACAGTAA
*Hprt1*	NM_012583.2	TTGTGTCATCAGCGAAAGTGG	CACAGGACTAGAACGTCTGCT

Abbreviations: *MafA*, V-Maf musculoaponeurotic fibrosarcoma oncogene homolog A; *NeuroD*, neurogenic differentiation factor 1; *Syt5*, synaptotagmin 5; *Snap25*, synaptosome associated protein 25; *Cacna1a*, calcium voltage-gated channel subunit alpha 1a; *Cacnb1*, calcium voltage-gated channel auxiliary subunit beta 1; *Vamp2*, vesicle associated membrane protein 2; *Jnk*, c-Jun N-terminal kinase; *Hprt1*, hypoxanthine phosphoribosyltransferase 1.

**Table 2:** Top *MAPK8IP1* variants at ±100 Kb and their associations with T2D. Data obtained from the TIGER data portal.

ID	Reference Allele	Alternate Allele	Sample Size	*p*-Value
Diagram Diamante				
rs7115753	A	G	231,420	4.08 × 10^−9^
Diagram 1000G				
rs11038677	T	C	-	3.70 × 10^−5^
70K for T2D				
rs553011963	CGTT	AGTT	66,940	0.0013

**Table 3:** Association of top MAPK8IP1 variants across all datasets and traits included in the Type2 Diabetes Knowledge Portal.

Phenotype	*p*-Value	Beta	Odds Ratio	Sample Size
**rs7115753**				
Fasting glucose adj BMI	9.76 × 10^−51^	▼−0.0246		287,195
Fasting glucose	2.75 × 10^−13^	▼−0.0298		263,726
Type 2 diabetes adj BMI	1.37 × 10^−7^		▼0.9635	266,778
Type 2 diabetes	1.62 × 10^−7^		▼0.9635	1,436,100
**rs7115753**				
Fasting glucose adj BMI	2.45 × 10^−24^	▼−0.0185		287,195
Fasting glucose	2.371 × 10^−8^	▼−0.0212		277,462
Type 2 diabetes adj BMI	3.59 × 10^−7^		▼0.9570	266,778
Type 2 diabetes	0.00004		▼0.9813	1,436,100

## Data Availability

Publicly available datasets were analyzed in this study. These data can be found here: (https://www.ncbi.nlm.nih.gov/geo/query/acc.cgi?acc=GSE50398)/ accession number GSE50398.

## Supplementary Materials

The following supporting information can be downloaded at: https://www.mdpi.com/article/10.3390/metabo13020307/s1, Figure S1: Bulk tissue expression for MAPK8IP1 (ENSG00000121653.11); Figures S2–S4 (replicas 1–3): The full-length Western blot expressions after *Mapk8ip1* silencing; Figure S5–S7 (replicas 1–3): the full-length Western blot expression after si*MafA* and si*Pdx1* silencing.
