# Supplementary material for: Expression Silencing of Mitogen-Activated Protein Kinase 8 Interacting Protein-1 Conferred Its Role in Pancreatic β-Cell Physiology and Insulin Secretion
Source: Metabolites. 2023 Feb 20;13(2):307. doi: 10.3390/metabo13020307 (PMC9964862; doi:10.3390/metabo13020307)
Supplement: Supplementary file 1 [file metabolites-13-00307-s001.zip › metabolites-2189883-supplementary.pdf]

Supplementary Figures

Figure S1

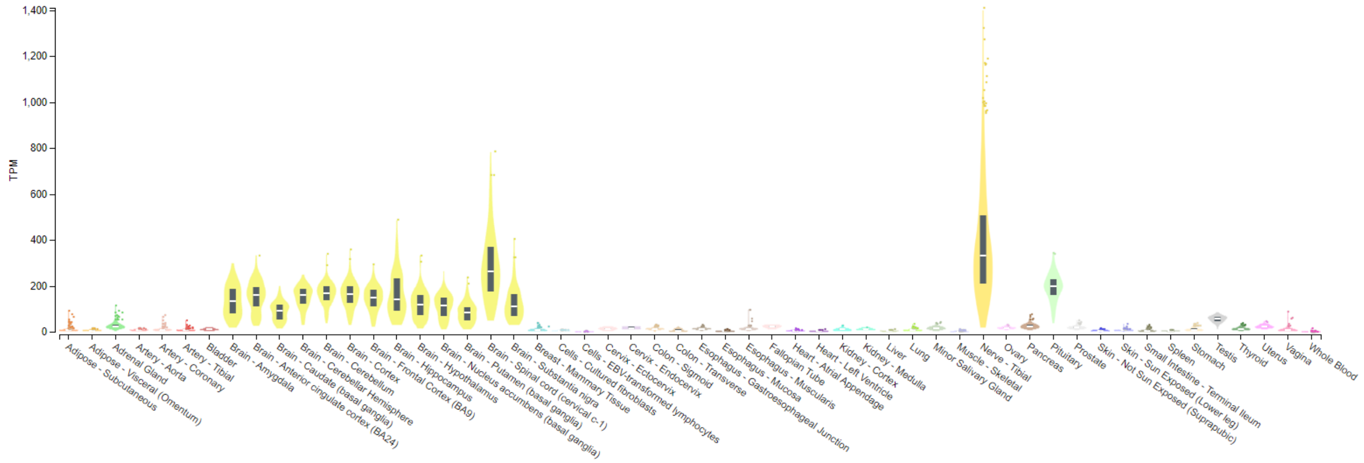

Supplementary Figure S1 Bulk tissue gene expression for MAPK8IP1 (ENSG00000121653.11).
Data Source: GTEx Analysis Release V8 (dbGaP Accession phs000424.v8.p2). Expression values are shown in TPM (Transcripts Per Million)

Figure S2 (replicate 1):

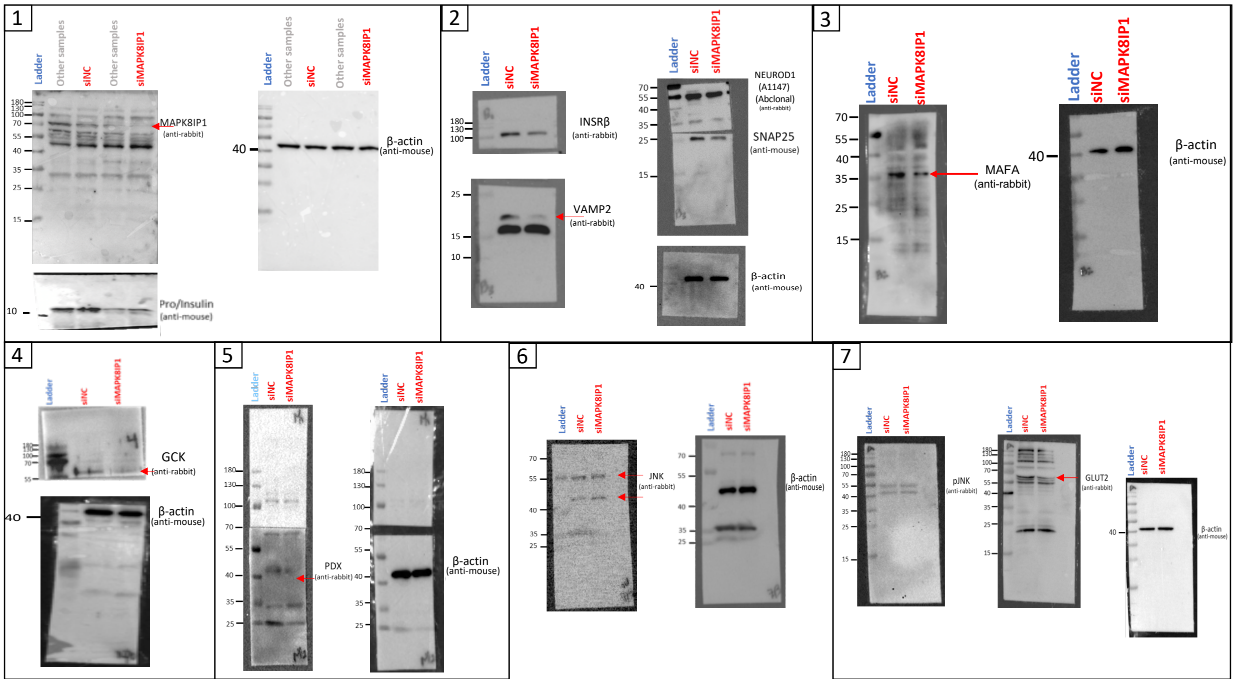

Supplementary Figure S2 (replicate 1) The full-length western blot expressions after *Mapk8ip1* silencing. Total protein was extracted 48 hours post-transfection. Western blot was conducted for **(1)** MAPK8IP1 and Pro/insulin, **(2)** INSRβ and VAMP2, NEUROD1 and SNAP25, **(3)** MAFA, **(4)** GCK, **(5)** PDX1, **(6)** JNK, **(7)** pJNK and GLUT2. β-actin was used as endogenous control. The cropped blots from membranes 2, 5 and 6 were used as representative figures for INSRβ, VAMP2, JNK, pJNK and GLUT2, respectively in the main article.

Figure S3 (replicate 2):

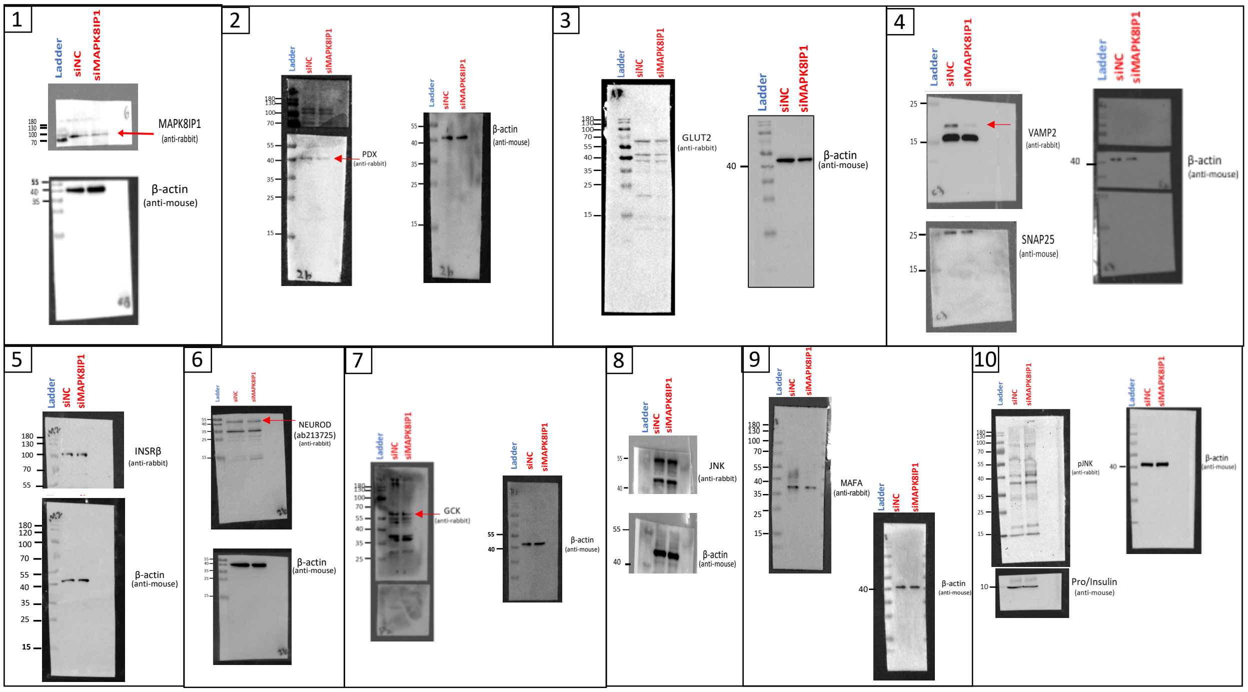

Supplementary Figure S3 (replicate 2) The full-length western blot expressions after *Mapk8ip1* silencing. Total protein was extracted 48 hours post-transfection. Western blot was conducted for **(1)** MAPK8IP1, **(2)** PDX-1, **(3)** GLUT2, **(4)** VAMP2 and SNAP25, **(5)** INSRβ **(6)** NEUROD1, **(7)** GCK, **(8)** JNK, **(9)** MAFA, **(10)** pJNK and Pro/insulin. β- actin was used as endogenous control. The cropped blots from membranes 4 and 8 were used as representative figures for SNAP25 and JNK respectively, in the main article.

Figure S4 (replicate 3):

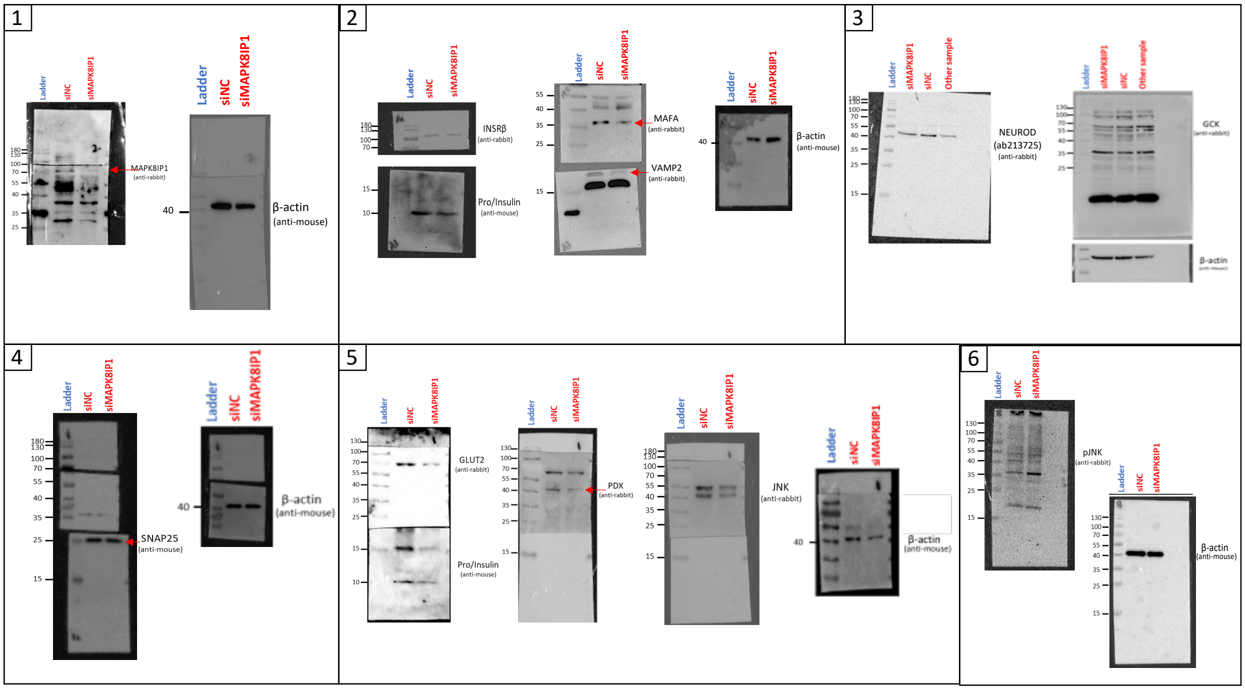

Supplementary Figure S4 (replicate 3) The full-length western blot expressions after *Mapk8ip1* silencing. Total protein was extracted 48 hours post-transfection. Western blot was conducted for **(1)** MAPK8IP1, **(2)** INSRβ, Pro/insulin, MAFA and VAMP2, **(3)** NEUROD1 and GCK, **(4)** SNAP25, **(5)** GLUT2, Pro/insulin, PDX1 and JNK, **(6)** pJNK. β-actin was used as endogenous control. The cropped blots from membranes 1, 2, 3 and 5 were used as representative figures for MAPK8IP1, MAFA, NEUROD1, GCK, Pro/insulin and PDX1, respectively in the main article.

Figure S5 (replicate 1):

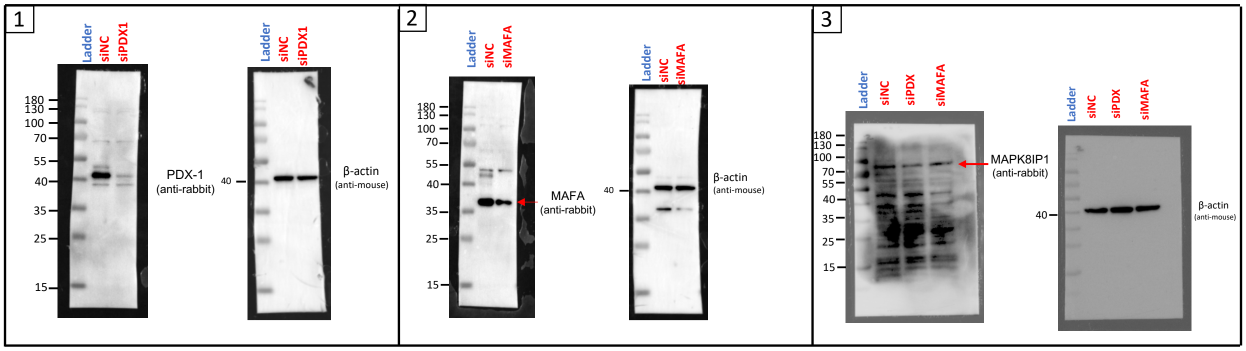

Supplementary Figure S5 (replicate 1) The full-length western blot expressions after *Pdx1* and *MafA* silencing. Total protein was extracted 48 hours post-transfection. Western blot was conducted for **(1)** PDX-1, **(2)** MAFA and **(3)** MAPK8IP1. β-actin was used as endogenous control. The cropped blots from membranes 1, 2 and 3 were used as representative figures in the main article.

Figure S6 (replicate 2):

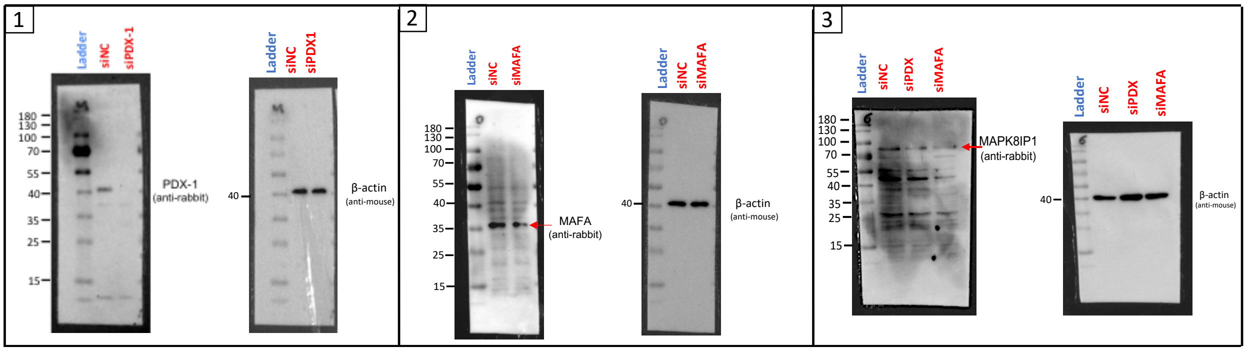

Supplementary Figure S6 (replicate 2) The full-length western blot expressions after *Pdx-1* and *MafA* silencing. Total protein was extracted 48 hours post-transfection. Western blot was conducted for **(1)** PDX-1, **(2)** MAFA and **(3)** MAPK8IP1. β-actin was used as endogenous control.

Figure S7 (replicate 3):

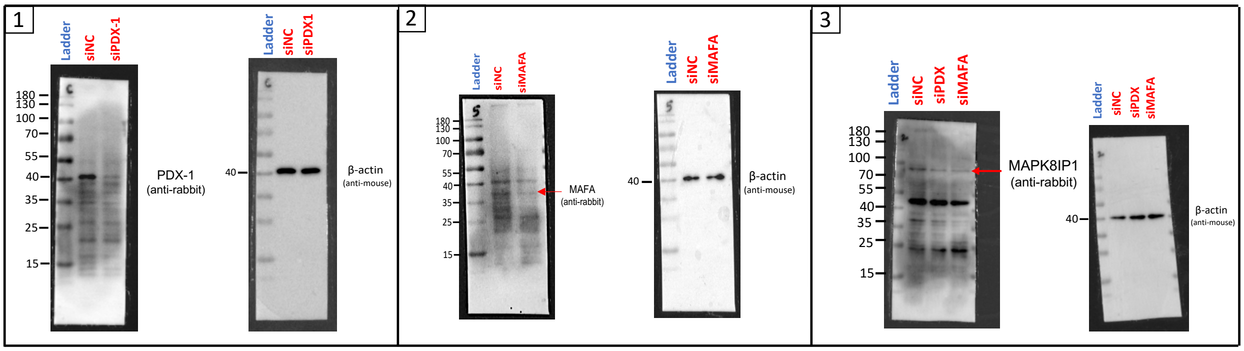

Supplementary Figure S7 (replicate 3) The full-length western blot expressions after *Pdx1* and *MafA* silencing. Total protein was extracted 48 hours post-transfection. Western blot was conducted for **(1)** PDX-1, **(2)** MAFA and **(3)** MAPK8IP1. β-actin was used as endogenous control.
